# Increasing the Use of Urban Greenways in Developing Countries: A Case Study on Wutong Greenway in Shenzhen, China

**DOI:** 10.3390/ijerph14060554

**Published:** 2017-05-23

**Authors:** Yiyong Chen, Weiying Gu, Tao Liu, Lei Yuan, Mali Zeng

**Affiliations:** 1 School of Architecture & Urban Planning, Shenzhen University, Shenzhen 518060, China; chenyiy@szu.edu.cn (Y.C.); yuanlei@szu.edu.cn (L.Y.); 2160140613@email.szu.edu.cn (M.Z.); 2 Shenzhen Key Laboratory of Built Environment Optimization, Shenzhen University, Shenzhen 518060, China; 3 Pingshan Center for Urban Planning & Land Affairs of Shenzhen, Shenzhen 518118, China; guweiy@163.com; 4 College of Urban and Environmental Sciences, Peking University, Beijing 100871, China

**Keywords:** frequency of use, duration of use, logistic regression, urban greenway

## Abstract

Given the benefits of urban greenways on the health and well-being of urban populations, the increased use of urban greenways has garnered increasing attention. Studies on urban greenways, however, have been mostly conducted in Western countries, whereas there is limited knowledge on greenway use in urban areas in developing countries. To address this shortcoming, the present study selected Wutong Greenway in Shenzhen, China, as a case study and focused on the use pattern and factors that influence the frequency and duration of urban greenway use in developing countries. An intercept survey of greenway users was conducted, and 1257 valid questionnaires were obtained. Multiple logistic regression analysis was used to examine the relationship between potential predictors and greenway use. Results showed that visitors with a varied sociodemographic background use Wutong Greenway with high intensity. Various factors affect the use of urban greenways, including individual and environmental factors and greenway use patterns. Unlike previous studies, we found that accommodation type, length of stay at present residence and mode of transportation to the greenway are important factors that affect greenway use. In contrast with studies conducted in Western countries, less-educated and low-income respondents visit the Wutong greenway even more frequently than others. Thus, the greenway is an important public asset that promotes social equity and that all residents can freely use. To better serve citizens, we suggest that the greenway network should be extended to other areas and that its environmental quality should be improved.

## 1. Introduction

Around the world, more and more people are living in cities and using urban infrastructure as a result of rapid urbanization. According to a statistical report from the World Health Organization, more than half of the world’s population lived in urban areas by 2010. In China, a developing country, 55.9% of the population lived in urban areas by 2015; this proportion continues to increase at a rapid pace [1]. Population density is extremely high in metropolitan areas of China, such as the Pearl River Delta area. As a result of rapid urbanization, developing countries experience many urban problems, including traffic congestion, environmental degradation and lack of public space.

Urban environments have an increasingly important role in the daily lives of urban residents. The growth of urban infrastructures is expected to accelerate over the next few years [2]. Green urban infrastructures, including parks, gardens, greenways, forests, wetlands, agricultural fields, green roofs, green walls, and all other kinds of urban green spaces, are an essential part of the urban system that serves the interests of both humans and nature [3]. Green infrastructures that support healthy behaviors are believed to have more permanent and population-wide effects than other forms of public health interventions [4]. The importance of green spaces to urban residents is also highlighted in urban planning policies as shorter distance to green space is associated with more use. Numerous studies have mentioned the importance of increasing the use of green space given the positive effect on supporting public health and providing social well-being for urban residents [5,6,7].

As urban green spaces, greenways have received increased attention over the past few years [8]. A greenway is a “linear open space established either along a natural corridor, such as a riverfront, stream valley, ridgeline, or overland along a railroad right-of-way that has been converted to recreational use, a canal, a scenic road, or other route” [9]. As a linear green infrastructure, greenways benefit everyone regionally and globally, thus this overcomes the issue of inequity and promotes social equity [5,7]. Numerous studies have illustrated the multiple functions of greenways as ecologically-significant corridors, recreational space and historically- or culturally-significant paths [10,11]. Since the 1980s, greenway planning projects have boomed in Western countries as planning practices with numerous ecological and social benefits. Since the new millennium, similar projects have spread to developing countries, such as China, as a part of the International Greenway Movement [8]. However, according to a recent study, only a quarter of the newly-built greenway network in China support activities, and three quarters of greenways cannot support physical activities, thus causing a significant waste of public resources [12]. Studies on the topic of greenways in China mainly introduce the planning and construction of Western greenways. Several studies have evaluated the ecological benefits of greenway in China [13,14], but the patterns of and the factors that influence greenway use in China remain unclear.

Studies have discussed the relationship between greenways and their usage and have reported that greenway location, length, width, pavement, facilities, accessibility and surrounding features, such as residential density and land use mixture, likely influence greenway use [10,15,16]. However, specific results vary. Gobster found that the distance between the visitors’ place of residence and the greenway strongly influences how a greenway is used, who uses it and how often it is used [16]. Several studies have shown that greenway users are more likely to be better educated and have higher incomes than the populations of the area where greenways are located [17]. Greenway users are likely to be young, male and never married; they are also likely to engage in transportation and physical activities and access the greenway via active transit modes [18].

Greenway use intensity and patterns vary considerably by trail segments [17] and are especially heavy in greenway sections that intersect parks and in downtown areas [19]. Furthermore, urban greenway infrastructure can effectively encourage high-density residential and commercial development. Appealing amenities in the greenway attract people and firms and thus promote property development [20]. Greenway use varies between different times in a day and between weekdays and weekends [21,22]. Daily outdoor physical activity on urban greenways is also significantly affected by weather conditions, such as daily maximum temperature, precipitation and wind speed [23,24]. Lighting, drinking water and restroom facilities, design, cleanliness, safety and parking lot availability are important factors related to the duration of greenway use [10]. Greenways with dense residences, mixed land use, an advanced street network and large parks support physical activities; furthermore, advanced public transportation further improves the diversity of greenway activity [12]. These studies provide useful insights into the nature of urban greenway use.

Although previous studies have provided useful information about the use pattern of urban greenways in Western countries, we know very little about the use of urban greenways in developing countries, especially about the use of newly-built urban greenways in China. Over the past few years, rapid urbanization in China has led to the construction of many public green spaces in high-density urban areas; many of these areas, however, are poorly used [25]. To date, the majority of the world’s population is concentrated in developing countries. Thus, the core battlefield of the greenway movement has moved to developing countries. Considerable attention should be given to user perception and factors that affect urban greenway use in developing countries. Considering the different developmental level and enormous cultural differences between Eastern and Western societies, research results for Western countries are not automatically valid for Eastern countries [26]. In addition, although individual and environmental factors that affect greenway use have been deeply discussed in previous studies, using pattern factors, such as the mode of transportation to the greenway and activities on the greenway, that may affect the frequency and duration of greenway use is still understudied [10].

The present study addresses this knowledge gap and attempts to identify the factors that influence the use of urban greenways in China. The aims of this study are: (1) to determine the use pattern of greenways in a high-density urban area of China; (2) to analyze the individual, environmental and greenway-use pattern factors that influence the frequency and duration of urban greenway use; and (3) to compare the differences in use pattern between developing and developed countries.

## 2. Methodology

### 2.1. Study Case

Greenways were first introduced in China as a type of spatial network based on the broad concept of the greenway network. As a result, Chinese greenways were developed with a systematic, large-scale and network-oriented view [12]. From 2010, the greenway network in the Pearl River Delta began to develop in accordance with a typical top-down process, which was designed as the network framework that connects regional country parks, nature preserves, historical heritage sites and cities.

**Figure 1:**
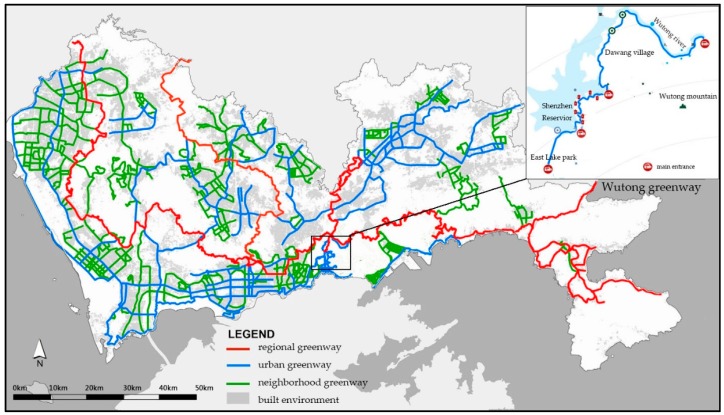
Shenzhen Greenway map and location of Wutong Greenway (base map from [31]).

The Shenzhen Greenway, a part of the Pearl River Delta Greenway, has been gradually constructed since its initial planning in 2010. The Shenzhen Greenway has a total length of more than 2370 km, by the end of May 2015 [27], and is structured on the basis of three greenway hierarchies: regional (343 km), urban (862 km) and neighborhood (1173 km). Shenzhen Greenway Planning is one of the first innovative planning projects in China. This project began in 2009 and currently covers most of the urban built-up areas of Shenzhen (Figure 1).

We selected Wutong Greenway in the Luohu district of Shenzhen, China, as the case study. Shenzhen City has a warm, monsoon-influenced, humid subtropical climate that is highly suitable for outdoor activities [28]. The long-term average annual temperature in this area is 23.0 °C with an average maximum temperature of 28.9 °C in July and an average minimum temperature of 15.4 °C in January. The average annual precipitation in this area is approximately 2000 mm. Rainfall exceeds 50 mm or more for approximately 10 days per year during the past 50 years [29]. Some precipitation is delivered by typhoons that strike from the east during summer and early autumn. Most of the year, however, the weather is fair and suitable for outdoor activity [30].

**Figure 2:**
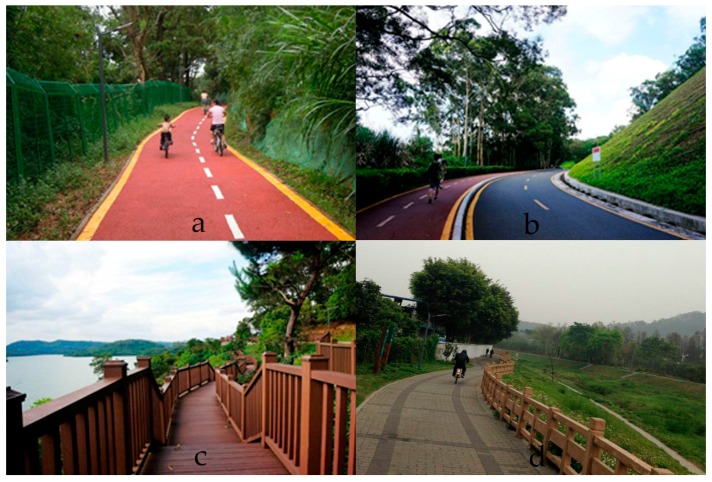
Photographs showing Wutong greenway: (**a**) greenway crossing the forest; (**b**) greenway along the road; (**c**) greenway besides the Shenzhen reservoir; (**d**) greenway along the Wutong river.

Wutong Greenway is a newly-constructed, well-equipped and well-maintained urban greenway with beautiful scenery (Figure 1 and Figure 2). It begins from the East Lake Park of Shenzhen, winds along the east bank of Shenzhen Reservoir and the upper area of the Wutong River, crosses Dawang Village (a densely-populated residential urban village) and ends at the connection of roads that climb to Wutong Mountain. The terrain of Wutong Greenway is rugged where it crosses mountainous areas, with multi-slopes. The Greenway is approximately 3 m wide and approximately 15 km long. Five main entrances, namely East Lake Park Entrance, Liantang Entrance, Xianhu Entrance, Dawang Entrance and Wutong Mountain Entrance, connect the greenway with the urban residential area. Wutong Greenway was built with a total investment of approximately 69 million yuan during 2013 and 2014 and was open to the public by the end of 2014. Since its opening, it has become a popular public destination for residents all over the city and has been praised as the most beautiful greenway in Shenzhen. Although only approximately 50,000 residents live within 1000 m of Wutong Greenway, the estimated total number of visitors to Wutong Greenway in 2016 is 1.11 million [32].

We selected Wutong Greenway as the case study for three main reasons: (1) visitors to Wutong Greenway are numerous and various; thus, we can analyze different use patterns and influential factors; (2) as a newly-built greenway, we can easily estimate the use situation of Wutong Greenway for comparison with those of other greenways; and (3) Wutong Greenway is located away from urban traffic roads; thus, it is a real greenway rather than a city sidewalk.

### 2.2. Questionnaire

In this study, questionnaires were used to collect data from the active users of Wutong Greenway. An intercept survey of greenway users was conducted at three key access points in November 2016. Greenway users aged 12 years or older and who passed the sampling location were randomly approached by a trained investigator and asked if they were willing to participate in a brief questionnaire on greenway use. Following acceptance, the on-site survey took approximately 5 min to complete. In total, 1300 questionnaires were handed out, 1297 questionnaires returned and 1257 (96.7%) considered as valid. A total of 40 invalid questionnaires was rejected because more than one choice was selected in response to single-choice questions or they were from respondents who were simultaneously under the age of 15 and married or have a child, which is not permitted by law in China.

The questionnaire was inspired by several other previous studies on the use of urban greenways [10,17] and includes two parts. The first part of the questionnaire consisted of general questions about greenway use. The respondents were first asked to estimate the distance from their place of residence to the entrance of the greenway. The possible answer categories were as follows: less than 500 m, 500 m–1 km, 1 km–3 km, 3 km–5 km and more than 5 km. The respondents were also queried about their mode of transportation to the greenway (walking, bicycle, public transportation and car); the frequency of greenway use (daily, several times per week, weekly, monthly and seldom); the duration of greenway use (<15, 15–30, 30–60, 60–120 and >120 min); activities on the greenway (jogging, cycling, brisk walking, slow walking, and others); and companion (single, lover, family, friend and pet). Satisfaction level on greenway use was rated on a five-point Likert scale [33,34]: extremely satisfied, moderately satisfied, neutral, unsatisfied and extremely unsatisfied. Then, respondents were asked about their main reasons for visiting the greenway. For this question, more than one option could be selected from the following: passing by, sports, reduce stress, lose weight, enjoy fresh air, social, family gathering, and others. The last question of the first part asked respondents about their thoughts on factors that influence greenway use (e.g., distance from their place of residence, accessibility, scenic view, terrain, lighting, restroom facilities, maintenance, trail width, safety, informative signs and parking lot). The respondents ranked their responses on a Likert five-point scale that ranged from “merely important” to “very important”.

The second part of the questionnaire dealt with the respondents’ demographic background. The respondents answered selected questions about their gender (male, female); age (<15 (children), 15–34 (youth), 35–50 (midlife), 50–64 (middle aged) and >65 (aged)); marital status (married and single); educational level (junior school or less, high school, college or postgraduate); job status (employed, self-employed, unemployed, retired or student); income in yuan/per month (6.9 yuan = 1 dollar or 7.5 yuan = 1 Euro in December 2016) (<2000, 2000–5000, 5000–8000 or >8000); accommodation type (rent, dormitory or collective dormitory and own housing); length of stay at their current residence (<1 year, 1–2 years and >2 years); car ownership (yes/no); whether they have a child under 6 (yes/no); and pet ownership (yes/no). In terms of age, children were excluded in regression, for most of them come with their family rather than on their own wish. Because the proportion of the aged in Shenzhen was too small (1.8%), the middle-aged and aged groups were merged as one group for convenience of multinomial logistic regression. In terms of educational level, although the education group was always in correlation with the age group [27], the education level is lower than that in developing countries [35]; 48.3% of the population of Shenzhen City has an educational level of junior school or less, 27.3% of high school and 24.5% of college or higher. Thus, it is worth exploring different use patterns between different educational levels.

According to the sociodemographic statistics (Table 1), the gender and age of the 1257 respondents represent those of the population of Shenzhen City. Approximately 53.8% of the respondents are male, 49.1% of whom are aged between 15 and 34 years old and 34.4% of whom are aged between 35 and 50 years old. Approximately 53.6% of Shenzhen residents are male, 51.9% of whom are aged between 15 and 34 years old and 30.7% of whom are aged between 35 and 50 years old [27]. The educational levels of the respondents, however, are not consistent with that of the population in Shenzhen City: approximately 19.6% of the respondents have an educational level of junior school or less, 37.2% of high school and 43.2% of college or higher. This difference may be partly because our questionnaires were sent only to adults. Regarding marital status, 70.2% of the respondents are married vs. 58.1% of Shenzhen residents [36]. In terms of monthly income level, the majority earns 5000–8000 yuan/month, compared with the average income of 6753 yuan per month in Shenzhen in 2015, according to the 2016 Shenzhen statistical yearbook. The main sociodemographic data of our respondents and the government statistical data are generally consistent. Therefore, the data of this study are representative of the population of Shenzhen to some extent.

Furthermore, data statistics (Table 1) revealed that of all the respondents, 85.6% are employed or self-employed, 14.0% are unemployed or retired and 50.0% are living in their own house; whereas 34.8% are living in a rental house and 15.3% are living in a collective dormitory. Up to 50.3% of respondents have stayed at the present residence for more than two years. Moreover, 45.1% of greenway users own a car; 20.4% have a child under 6; and 27.6% keep a pet.

### 2.3. Model Construction and Data Analysis

To identify the factors that affect the frequency and duration of greenway use, multinomial logistic regression models [37] were constructed as shown in Equation (1):

In(π*_j_*/π*_J_*) = a*_j_* + *β_j_*_1_X_1_ + … + *β_jk_*X*_k_* + … + *β_jp_*X*_p_*, *j* = 1, …, *J*−1 (1)

where π represents the frequency or duration of greenway use of the sample *i*; a*_j_* is a constant term; X*_k_* represents potential predictors; *J* represents the categories of π; *p* represents the number of predictors; and *β* represents regression coefficients.

Potential predictors include individual factors, such as gender, age, marital status, educational level, job status, income level, accommodation type, length of stay at present residence, vehicle ownership, children under 6 and pet ownership, and greenway use patterns, such as distance from place of residence to greenway entrance, accessibility, mode of transportation to the greenway, whether they have a companion and activities on the greenway. The results are presented as the odds ratio (OR), sig. and χ-square, *p*-value. Goodness-of-fit of the models is assessed by the χ-square test, and the tests indicated that the models fit the data adequately. A *p*-value of 0.05 was used to indicate statistical significance. SPSS Version 22 (IBM, Armonk, NY, USA) was used for all statistical analyses.

## 3. Key Findings

### 3.1. Distance between Home and Greenway and Patterns of Use

#### 3.1.1. Distance between Place of Residence and the Greenway, Mode of Transportation, Frequency of Use and Duration of Use

As shown in Table 2, of all of the respondents, 29.3%, 59.0% and 18.5% of the respondents reside within 500 m, 3 km or 5 km away from the entrance of Wutong Greenway, respectively. When asked about their mode of transportation to the greenway, 80.2% of the respondents provided “walk” or “bicycle”, and only 19.8% answered “public transportation” or “car”. In terms of the frequency of use, 64.8% visited Wutong Greenway daily or several times a week, and 6.9% visited Wutong Greenway monthly or infrequently. In terms of the duration of use, 47.5%, 35.1% and 1.5% of the participants spent more than 60 min, 30–60 min or less than 15 min at the greenway.

#### 3.1.2. Relationship between Distance from the Greenway and Greenway Use

**Figure 3:**
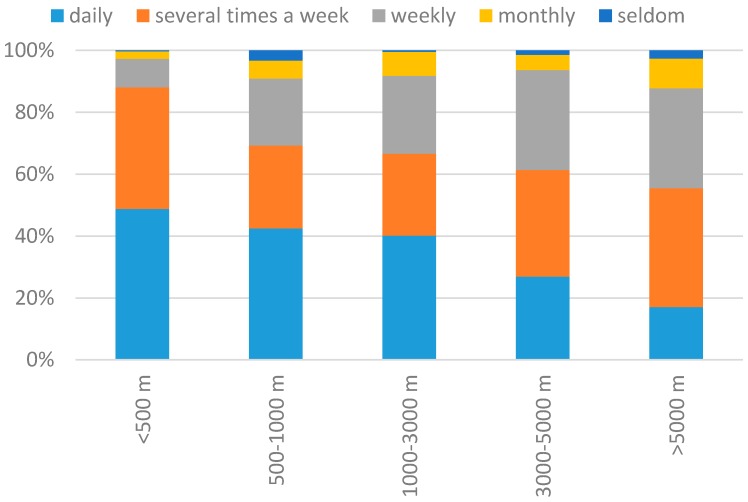
Distance to the Wutong Greenway vs. frequency of use, in percent of the respondents.

As shown in Figure 3, the frequency of visiting Wutong Greenway decreases as the distance from home to the greenway entrance increases. The proportion of daily visitors decreases from 39.0% among visitors who live within 500 m of the greenway and to 17.0% among visitors who live over 5000 m away from the greenway. Monthly and infrequent visitors increase from 2.2% among visitors who live within 500 m of the greenway to 12.2% among visitors who live over 5000 m away from the greenway.

**Figure 4:**
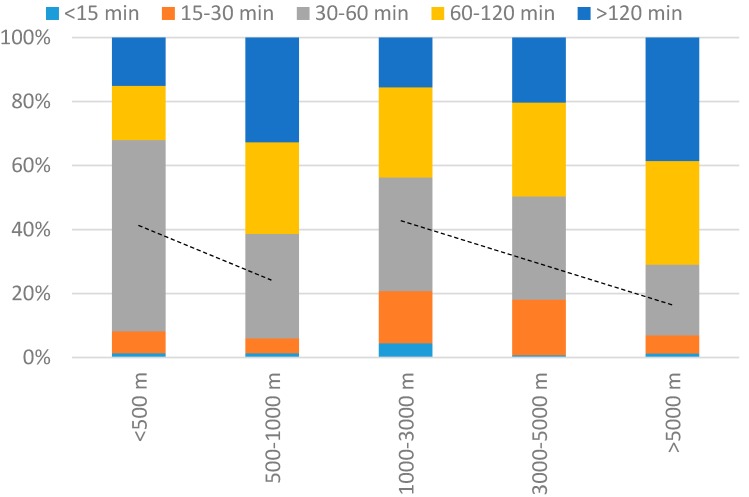
Distance to Wutong Greenway vs. duration of use, in percent of the respondents.

As shown in Figure 4, the duration of greenway use increases slightly as the distance from home to the greenway entrance increases. Two different use modes are detected. When the distance between their homes and the greenway is within 1000 m, the majority of visitors stay for a short time, and the duration of greenway use increases as distance increases. When the distance between their homes and the greenway is over 1000 m, the majority of visitors stay for more than 1 h, and the duration of greenway use increases as distance increases. The former could be defined as neighborhood users, who come to the greenway more frequently, whereas the latter could be defined as regional users who come to the greenway less frequently, they stay longer as distance increases.

#### 3.1.3. Behavior and Patterns of Use

The main activities in the Wutong Greenway include jogging, cycling and walking. Other activities are seldom observed or received as answers from the respondents. Of all of the respondents, 36.7% visit the greenway by themselves, and 48.8% are with a lover or their family. Interestingly, our data analysis pointed out that approximately half of the unmarried visitors came with a lover. Only 1.6% of respondents visit with a pet. Respondents visit the greenway mainly for recreational and health purposes. Of these visitors, 66.0% visit the greenway to engage in sports, 42.7% to enjoy fresh air, 18.1% to lose weight and 18.5% to reduce stress. Some visitors come mainly for social reasons, including 4.0% who visit for social reasons and 2.5% for family gatherings. The results also revealed a high satisfaction level on the use of Wutong Greenway, with 43.6% of visitors being extremely satisfied and 44.8% being moderately satisfied. Only 0.2% of the respondents are unsatisfied or extremely satisfied (Table 3).

Parking lot, trail width and safety are the three most important perceived factors that affect the use of this scenic greenway. Considering the conditions of Wutong Greenway, these findings are not surprising: there is only one small parking lot at one entrance of the greenway, whereas 11.3% of the participants arrived at the greenway via car. Moreover, 45.1% of the respondents own a car. The width of the greenway is approximately three meters. Given its mixed use by walkers, runners and cyclists, the narrow width of the greenway is a considerable safety problem. The factors that affect the frequency and duration of greenway use are discussed further in the next section.

### 3.2. Factors That Influence the Frequency of Greenway Use

Multinomial logistic regression analysis was performed with “using the greenway at least twice a week” as a dependent factor and with sociodemographic attributes and greenway use pattern variables as potential predictors. The results (Table 4) revealed that the odds of using the greenway at least twice a week are significantly affected by both individual factors (including age, marital status, educational level, income level, accommodation type and length of stay at present residence) and greenway use pattern factors (including distance from home and mode of transportation to the greenway). The frequency of visiting the greenway increases with age. In terms of education, visitors with low educational levels (junior school or less) visit the greenway more frequently than those with higher educational levels (high school or college). In terms of income level, those with low income visit the greenway more frequently than those with high income. The length of stay at present residence also affects the frequency of greenway visits; respondents who have stayed at their present residence for less than a year visit the greenway more frequently than those who have stayed at their present residence for more than two years (OR = 2.07, 95% CI: 1.16–3.67). Moreover, respondents who live in rental accommodations visit the greenway less frequently than those who live in their own house (OR = 0.50, 95% CI: 0.30–0.83).

Our data analysis (Table 4) also reveals that the frequency of greenway visits significantly decreases as the distance from home to the greenway entrance increases. Respondents who live within 3000 m from the greenway entrance (<500 m, OR = 1.90, 95% CI: 1.35–2.68; 500 m–1000 m, OR = 1.81, 95% CI: 1.18–2.79; 1000 m–3000 m, OR = 1.61, 95% CI: 1.01–2.36) are more likely to visit the greenway more frequently than those who live over 5000 m away. In terms of the mode of transportation to the greenway, respondents who walk to the greenway are more likely to visit the greenway more than twice a week than those who arrive by car (OR = 3.19, 95% CI: 2.01–4.92). Respondents who arrive by public transportation visit the greenway less frequently than those who arrive by car (OR = 0.52, 95% CI: 0.30–0.93). Regression reveals that activities on the greenway and the presence of a companion do not affect visiting frequency.

### 3.3. Factors That Influence the Duration of Greenway Use

To obtain a better understanding of the factors that affect the duration of the use of Wutong Greenway, an additional multinomial logistic regression analysis was performed with “time spent on the greenway for more than an hour” as a dependent factor. The results (Table 4) revealed that the odds of spending more time on the greenway are significantly affected by both individual factors (including gender, age, marital status, education level, job status, income level, accommodation type, length of stay at present residence and pet ownership) and greenway-use pattern factors (including distance from present residence, activities, companion during greenway visit and mode of transportation to the greenway). In detail, married, female respondents, aged 50 years old are significantly more likely to spend more time at the greenway than single, male respondents, aged between 35 and 50 years old. Moreover, individuals with a low educational level are likely to spend a long time on the greenway. Compared with respondents with postgraduate degrees, the odds of spending more time on the greenway for respondents with an educational level of junior school or less are 4.85 (95% CI: 1.93–12.19); those for respondents with an educational level of high school are 8.043 (95% CI: 3.76–17.20); and those for respondents with an educational level of college are 2.86 (95% CI: 1.43–5.71). In terms of job status, self-employed (OR = 1.59, 95% CI: 1.10–2.30), unemployed (OR = 20.98, 95% CI: 4.04–108.83) or retired (OR = 3.00, 95% CI: 1.56–5.77) respondents are more likely to spend more time than employed visitors. In terms of income level, low-income visitors are more likely to spend more time at Wutong Greenway than high-income (monthly income of more than 2000 yuan) visitors. In addition, visitors who reside in rental accommodations (OR = 2.06, 95% CI: 1.22–3.47) or a collective dormitory (OR = 2.68, 95% CI: 1.43–5.00) are more likely to spend more time at the greenway than respondents who live in their own house. Furthermore, results showed that respondents who have stayed at their current residence for a long period are more likely to spend more time at the greenway. Moreover, respondents who own a pet are more likely (OR = 1.56, 95% CI: 1.01–2.21) to spend more time at Wutong Greenway than those who do not have pets.

Regression (Table 4) also reveals that for respondents who live 1000 m away from the greenway entrance, the odds of spending more time on the greenway increase with increasing distance from home to the greenway entrance. Visitors who walk slowly on the greenway are more likely to spend more time on the greenway than those who are jogging, cycling or brisk walking. Respondents with friends are more likely to spend more time than single visitors or visitors with lovers. In terms of transportation mode to the greenway, respondents who arrive by car (OR = 2.49, 95% CI: 1.45–4.26) or by public transport (OR = 3.02, 95% CI: 1.76–5.16) are more likely to spend more time on the greenway than those who walk to the greenway.

## 4. Discussion

### 4.1. Patterns of Greenway Use

In the present study, we selected Wutong Greenway in Shenzhen to explore the use pattern of greenways in a high-density urban area in China and to analyze the factors that influence the frequency and duration of urban greenways’ use. We found that Wutong Greenway is used by visitors with various sociodemographic attributes at a high intensity. Greenway use is affected by both individual, environmental and greenway-use pattern factors. In general, our findings are consistent with those of previous studies [10,12,18].

Regarding the frequency and duration of use, 64.8% of respondents visit Wutong Greenway more than once a week. Furthermore, 47.5% of these visitors spend more than 1 h on the greenway, whereas 35.1% spend 30–60 min. Schipperijn et al. reported that 72.9% of Danes visit an open space more than once a week [38]. Sanesi and Chiarello reported that 26% and 60% of Italians use urban green spaces several times or once a week, respectively [39]. In another study, 90.7% of respondents visit a greenway more than once a week, whereas 52.5% and 35.3% use greenways for more than 1 h or 30–60 min, respectively [10]. In comparison, the usage frequency and duration of each visitor to Wutong Greenway, however, are not as high as its kind.

We found that Wutong Greenway users are more likely to be male, youths, married, employed, have resided at their present residency for a long time and are living in their own house; these results reinforced those of previous studies of developed countries [18,40] and China [22,41]. Comparing with Akpinar’s study, our data statistic reveals a higher proportion of low-income visitors and employed visitors, a lower proportion of retired visitors and well-educated visitors on Wutong Greenway [10]. This difference mainly comes from the sociodemographic attribute characteristics of Shenzhen City as stated at Section 2.2. Thus, it is suggested that greenway built should be consistent with the demographic structure and different needs. 

### 4.2. Factors That Affect Greenway Use

Our data analysis revealed that eight factors significantly affect both the frequency and duration of greenway use. These factors include individual factors (age, marital status, educational level, income level, accommodation type, length of stay at present residence) and greenway-use pattern factors (including distance from home and mode of transportation to the greenway). The factors that influence the frequency and duration of use are slightly different. Five more factors affect the duration of greenway use, including gender, job status, pet ownership, activities on the greenway and companion while visiting the greenway. Female, unemployed or retired, slow walkers and visitors with friends or a pet are more likely to spend more time on the greenway. In Western countries, individual factors, such as age, education, income and gender, are factors that affect the use of green space [38,42,43]. Owning a dog is a significant factor that increases the frequency of green space use in Denmark [44].

One important difference comparing with Western studies revealed by our findings is that accommodation type and length of stay at present residence are important factors that affect greenway use. Visitors who reside in rental accommodations or a collective dormitory use greenways less frequently, but are more likely to spend more time on the greenway than respondents who live in their own house. As China is experiencing rapid urbanization, population mobility between urban and rural area is very high, and numerous migrant workers live in rental houses or collective dormitories; the lifestyles of these migrants are different from those who live in their own houses and have stayed at their present residence for longer [45,46]. The rental house or collective dormitories usually are located in urban villages or old urban areas, where building environment is crowed and public open space is scarce. The residents have to go far away to use urban parks or greenways, with less usage frequency and a long duration. It is thus recommended that public open spaces be added in such areas.

As most studies [10,44,47] have revealed, distance is an important factor that affects the frequency and duration of greenway use. The frequency of visits to Wutong Greenway decreases as its distance from the home of the respondents increases. However, our findings revealed that only 41.5% of visitors to Wutong Greenway live within 1000 m from the greenway entrance and that 41.0% of visitors live 3000 m away from Wutong Greenway. In Akpinar’s study, 79.8% of greenway users live within 1000 m of the greenway [10]. In Schipperijn et al.’s study, 84.7% of residents live within 1000 m of green spaces [38]. The service area of Wutong Greenway is considerably larger than those of other greenways. Furthermore, Wutong Greenway is used in high intensity. Even though Shenzhen City has a huge greenway system, only a quarter of these greenways are active [12], and very few were built with high standards. More than half of Shenzhen Greenway occupies a former sidewalk. Its width is delineated by two lines. Moreover, it does not offer any attractions or facilities, thus attracting few and infrequent recreational users. The urban greenway system in China, with Shenzhen as a typical case, is far from perfect. The greenway network should be elaborated exquisitely and extended to more areas [11]. Furthermore, the environmental quality and affiliated facilities should be improved.

Another important factor we find is that respondents who walk or ride to the greenway visit the greenway more frequently than those who arrive by car or by public transportation. Visitors who arrive by car or public transportation, however, are more likely to spend more time on the greenway than visitors who walk or ride to the greenway. To the best of our knowledge, this factor is usually not discussed in previous studies. This finding is consistent with the different use patterns of Wutong Greenway. As distance between home and greenway increase, the travel mode gradually changes from walking and cycling to driving and public transportation. The identification of this factor is important in future efforts to increase urban greenway use. A convenient and friendly walking and cycling system connecting the urban greenway with neighborhood residential area will promote neighborhood residents to use greenway more frequently. Additionally, good public transportation system and enough parking lots could attract more users far away from the urban greenway.

In previous studies conducted in developed countries, public space users were proven more likely to be better educated and have higher incomes than the populations of the area where greenways are located [17,35,38]. However, our regressions revealed that less-educated and low-income respondents visit the greenway more frequently and spend more time at the greenway than well-educated and high-income respondents. As the public green space system in China is still not very systematic [48], both high-income and low-income residents are using limited green spaces with high intensity. Low-income groups that reside in urban villages have less access to urban green spaces in their neighborhoods. Thus, they use other urban green spaces at a high intensity. Therefore, as a distinct factor between developed countries and developing countries, the greenway could promote social equity in developing countries because all residents could freely visit and use the greenway [5], and the less-educated and low-income respondents visit the greenway even more frequently than others. More attention should be paid to understanding and acting to improve urban green spaces, living conditions, especially in the deprived areas, to promote social equity [49].

Three crucial environmental factors (problems) were perceived as important factors that affect greenway use: parking lot, trail width and safety. These factors are all main existing problems of Wutong Greenway and are similar to those that have been identified in studies in Western countries [10,39] and in other areas of China [22]. This result indicates that the problems that exist in developed countries also exist in developing countries when it comes to greenway conditions, as discussed in Akpinar’s study. The width of greenways in Pearl River Delta is approximately 2–3 m, while some are only 1.2 m. The affiliated facilities such as parking lot, drinking water and toilet are far from perfect. As Goličnik Marušić has pointed out, the physical spatial capacity and usability of a place play a key role in the relationship between places and their use [50]; thus, the environmental factors of the greenway are the physical basis for increasing urban greenway uses.

### 4.3. Future Perspectives

Our results show that different greenway-use patterns and factors affect the frequency and duration of urban greenway use. As a greenway with a total length of 15 km, the different use patterns in different parts of greenways require further exploration. Moreover, the use pattern of the surrounding land may also be a factor that affects greenway use, user amounts and density; this would be possible if user data at different times could be counted using automatic counters. Another limitation was that the self-reported measure of distance, time spent on greenway and visiting frequency may be subject to some issues related to recall error or estimate mistakes. Therefore, a combined method using questionnaires, observed data or other sources of data would be more accurate if possible.

A greenway is a relatively inexpensive and intensively-used public product that is provided by the government [5]. It is warmly welcomed by both high-income and low-income groups. Calculating a precise cost per visit could offer a useful reference for future policy makers because this could demonstrate that a greenway provides numerous benefits for a relatively small budget. The system of Shenzhen Greenway is hierarchically structured as regional, urban and neighborhood greenways, with different locations and construction models. The previous studies, as well as the current study, mainly focus on a certain kind of greenway [22,41]. Therefore, the exploration of the different use patterns among urban, regional and neighborhood greenways, is another interesting topic for future research.

## 5. Conclusions

Wutong Greenway is used by visitors of various socio-demographic attributes with high intensity. Various individual, environmental and greenway use pattern factors affect urban greenway use. Our findings are generally consistent with those of previous studies. However, we found that the type of accommodation, length of stay at present residence and mode of transportation to the greenway are important factors that affect greenway use.

Compared with studies in developed countries, less-educated and low-income respondents visit the Wutong Greenway even more frequently than others. Therefore, the greenway is an important public asset that promotes social equity because all residents can freely use the greenway in developing countries.

The frequency of visits to Wutong Greenway decreases as the distance between the greenway and the respondent’s home increases. However, the service area of Wutong Greenway is considerably larger than others, and the greenway is used with high intensity. The findings of the present study, therefore, suggest that to better serve more citizens, the greenway network should be extended to other areas, and its environmental quality should be improved. It is thus recommended that city planners and policy makers should continue to take distance to greenway into consideration, especially for deprived residential areas, in areas with many residents with limited public open spaces and in old urban areas. In existing neighborhoods, innovative solutions are needed as adding more public space is often impossible; a walking- and cycling-friendly built environment and convenient transportation system also promote the use of surrounding open spaces.

## Figures and Tables

**Table 1:** Sociodemographic characteristics of the study population.

Items	Choices	Results
Gender	Male	53.8%
Age	<15	3.7%
	15–34	49.1%
	35–50	34.3%
	>50	12.9%
Marital status	Married	70.2%
Educational level	Junior middle school or less	19.6%
	High school	37.2%
	College	36.1%
	Postgraduate	7.1%
Job status	Employed	63.0%
	Self-employed	22.6%
	Unemployed	2.7%
	Retired	11.3%
	Student	0.4%
Income level (yuan/per month)	<2000	3.4%
	2000–5000	26.0%
	5000–8000	34.3%
	>8000	36.3%
Accommodation type	Rent	34.8%
	Dormitory	15.3%
	Own housing	50.0%
Length of stay at present residence	<1 year	11.8%
	1–2 years	37.8%
	>2 years	50.3%
Vehicle ownership	Yes	45.1%
Have a child under 6	Yes	20.4%
Pet ownership	Yes	27.6%

**Table 2:** Distance, transportation mode, frequency and duration of the users.

Items	Choices	Results
Distance between home and the greenway	<500 m	29.3%
	500 m–1000 m	12.2%
	1000 m–3000 m	17.5%
	3000 m–5000 m	22.5%
	>5000 m	18.5%
Mode of transportation to the greenway	Walk	60.6%
	Bicycle	19.6%
	Public traffic	8.5%
	Car	11.3%
Frequency of greenway use	Daily	32.9%
	Several times a week	31.9%
	Weekly	28.3%
	Monthly	5.5%
	Seldom	1.4%
Duration of greenway use	<15 min	1.5%
	15–30 min	15.9%
	30–60 min	35.1%
	60–120 min	24.3%
	>120 min	23.2%

**Table 3:** Behavior of users in Wutong Greenway.

Items	Choices	Results
Activities	Jogging	39.3%
	Cycling	19.1%
	Brisk walking	25.2%
	Slow walking	16.2%
	Other	0.2%
Companion	Single	36.7%
	Lover	19.8%
	Family	29.0%
	Friends	12.9%
	Pet	1.6%
Reason for visit (multiple choice)	Engage in sports	66.0%
	Enjoy fresh air	42.7%
	Reduce stress	18.5%
	Lose weight	18.1%
	Passing by	5.3%
	Social	4.0%
	Family gathering	2.5%
	Other	0.5%
Satisfaction level of greenway use	Extremely satisfied	43.6%
	Moderately satisfied	44.8%
	Neutral	11.4%
	Unsatisfied	0.1%
	Extremely unsatisfied	0.1%
Perceived influencing factors for greenway use *	Distance from home	2.110
	Accessibility	2.474
	Scenic view	2.594
	Terrain	2.785
	Lighting	2.949
	Restroom facilities	2.880
	Maintenance	3.008
	Trail width	3.338
	Safety	3.326
	Informative signs	3.277
	Parking lot	3.354

* Five point range from 5 as “very important” and 1 as “very unimportant.

**Table 4:** Odds ratio for potential factors influencing greenway use.

Individual Factors	Categories	Frequency of Greenway Use				Duration of Greenway Use			
		OR	Sig.	Chi-Square (χ^2^)	*p*-Value	OR	Sig.	Chi-Square (χ^2^)	*p*-Value
Gender	Male	1.351	0.053			0.590	0.001		
	Female	1	-	3.741	0.053	1	-	11.503	0.000
Age	15–34	0.366	0.002			0.816	0.462		
	35–50	0.477	0.009			0.545	0.011		
	>50	1	-	1.909	0.012	1	-	1.088	0.018
Married	Yes (No = 1)	1.342	0.203	1.624	0.203	1.897	0.005	8.089	0.004
Education level	Junior or less	1	-	16.93	0.001	4.849	0.001		
	High school	0.442	0.001			8.043	0.000		
	College	0.492	0.014			2.856	0.003		
	Postgraduates	0.874	0.750			1	-	47.868	0.000
Job status	Employed	1	-	6.826	0.145	1	-	3.709	0.000
	Student	1.573	0.728			0.698	0.783		
	Self-employed	0.748	0.109			1.590	0.013		
	Unemployed	3.124	0.109			2.977	0.000		
	Retired	1.249	0.565			3.003	0.001		
Income level (yuan/per month)	<2000	9.440	0.000			15.676	0.000		
	2000–5000	4.175	0.000			2.476	0.001		
	5000–8000	1.309	0.157			5.292	0.000		
	>8000	1		47.819	0.000	1	-	92.150	0.000
Accommodation type	Rent	0.496	0.007			2.056	0.007		
	Dorm	0.779	0.431			2.676	0.002		
	Own housing	1	-	9.634	0.008	1	-	1.142	0.006
Length of stay at present residence	<1 year	2.065	0.014			1	-	16.938	0.000
	1–2 years	1.127	0.557			2.182	0.004		
	>2 years	1	-	7.156	0.028	3.481	0.000		
Have a car	No (yes = 1)	0.717	0.119	2.414	0.12	0.902	0.664	0.189	0.664
Have child under 6	Yes (No = 1)	0.758	0.164	1.929	0.165	0.747	0.127	2.331	0.127
Have a pet	Yes (No = 1)	0.772	0.159	1.985	0.159	1.556	0.014	6.095	0.014
Distance from home	<500 m	1.901	0.000			0.294	0.000		
	500–1000 m	1.811	0.007			0.634	0.099		
	1000–3000 m	1.606	0.015			0.115	0.000		
	3000–5000 m	1.275	0.179			0.437	0.001		
	>5000 m	1	-	16.641	0.002	1	-	77.812	0.000
Activities	Jogging	1.452	0.142			0.419	0.000		
	Cycling	1.191	0.511			0.364	0.000		
	Brisk walking	0.836	0.489			0.31	0.000		
	Slow walking	1	-	7.994	0.152	1	-	25.157	0.000
Companion	Single	0.685	0.112			0.248	0.000		
	Lover	1.027	0.925			0.202	0.000		
	Family	1.104	0.691			1.270	0.328		
	Friends	1		7.812	0.051	1	-	108.77	0.000
How to arrive	Walk	3.185	0.000			1	-		
	Bicycle	1.481	0.105			0.996	0.983		
	public traffic	0.523	0.026			3.017	0.000		
	Car	1	-	81.864	0.000	2.488	0.001	26.153	0.000
